# Occurrence of Tetrodotoxin in Bivalves and Gastropods from Harvesting Areas and Other Natural Spaces in Spain

**DOI:** 10.3390/toxins11060331

**Published:** 2019-06-11

**Authors:** Lucía Blanco, Jorge Lago, Virginia González, Beatriz Paz, Maria Rambla-Alegre, Ana G. Cabado

**Affiliations:** 1 ANFACO-CECOPESCA, Campus University 16, 36310 Vigo PO, Spain; lucia@anfaco.es (L.B.); jlago@anfaco.es (J.L.); virginia@anfaco.es (V.G.); beapaz@anfaco.es (B.P.); 2 IRTA, Institute of Agrifood Research and Technology. Ctra. Poble Nou km 5.5, 43540 San Carles de la Ràpita, Tarragona, Spain; maria.rambla@irta.cat

**Keywords:** tetrodotoxin, bivalves, gastropods, risk evaluation, LC-MS/MS

## Abstract

Tetrodotoxin (TTX) is a potent neurotoxin that is receiving increasing interest in the European Union because it has been found in different fishery products (fish, bivalves and gastropods) captured in European waters. Since available information is scarce, further analytical data regarding the incidence of this toxin in European fishery products is needed in order to perform an appropriate risk assessment devoted to protecting consumers’ health. Hence, samples of bivalves and gastropods were collected at different points of the Spanish coast and analyzed by high-performance hydrophilic interaction liquid chromatography-tandem mass spectrometry (HILIC-MS/MS) to evaluate the presence of TTX. None of the analyzed samples showed TTX above an internal threshold of 10 µg/kg or even showed a peak under it. Our results on TTX occurrence obtained in bivalve molluscs and gastropods did not show, at least in the studied areas, a risk for public health. However, taking into account previous positive results obtained by other research groups, and since we did not detect TTX in our samples, a more completed study increasing sampling frequency is needed to ensure proper risk evaluation towards the food safety of these products.

## 1. Introduction

Tetrodotoxin (TTX), a natural toxin exhibiting extreme neurotoxicity, has been found in many fish species, particularly puffer fish, globefish and toadfish, but also in arthropods, echinoderms, algae, molluscs, Nemertean worms and even in terrestrial animals such as amphibians. This toxin, considered a threat to human health in Asian countries, is restricted to warm water. However, it has been shown that the risk is also present in the Pacific and Mediterranean. Tetrodotoxin is a powerful neurotoxin which binds to the sodium channels of excitable cells. Intoxications are characterized by tingling of the tongue and lips, headache, vomiting, muscle weakness, ataxia and even death due to respiratory and/or heart failure (reviewed in [1]). As there is no antidote available, patients must receive ventilator and hemodynamic support in order to keep the patient alive in the first 24 h after intoxication [2].

The finding of TTX in European bivalve molluscs, together with a possible increase in sea surface temperatures of coastal waters, have triggered the need to monitor TTX, as identified by the European Emerging Risk Exchange Network (EREN) [3].

The origins of TTX remain controversial: bacteria from the genera *Vibrio*, *Alteromonas*, *Shewanella*, *Pseudomonas*, *Bacillus* and *Aeromonas*, among others, have been identified as causative agents of this potent toxin (reviewed in [1,4]). A possible link between the occurrence of the dinoflagellate *Prorocentrum minutum* and the detection of TTX in bivalves has been suggested [5]. Recent research has reported that TTX is linked to the presence of high contents of *Vibrio* in European coasts, namely *Vibrio parahaemolyticus*, in bivalves from the United Kingdom [6], and *Vibrio* spp. in bivalves from the northwest of Spain [7]. TTX was also detected in mussels and clams during a bloom of *Alexandrium minutum* and *Alexandrium pacificum* in Syracuse Bay (Sicily, Italy), along with an extremely high content of paralytic shellfish toxins (PSTs) [8]. This study points out the usefulness of a common method to quantify TTX, its analogues (TTXs) and PSTs, since both kinds of toxins are co-extracted under the same conditions. A combined detection may also possible, although further work is required to achieve this.

In the last years, TTX and its analogues have been reported in marine bivalves and gastropods from European waters, analyzed by liquid chromatography with tandem mass spectroscopy (LC-MS/MS). This methodology is the best option for the assessment needed, since LC-MS/MS methods are the most suitable for the identification and quantification of TTX and its analogues with adequate limits of quantitation (LOQs) [9].

In the European Union (EU), the scientific opinion performed by the EFSA concluded that there is no general concern for human health due to the ingestion of marine bivalves except in the case of the consumption of a large portion of oysters [9]. However, the EFSA recommends the evaluation of the presence of TTX in bivalves and gastropods from EU waters in order to perform an appropriate risk evaluation. TTX is not a regulated toxin in European countries at present, although according to the current EU legislation, fish belonging to the poisonous family of Tetraodontidae or products derived from them must not be placed on the European markets [10,11]. In contrast, Japan and Korea apply guidelines and legislation regarding TTX in this fish species, with 2 μg of TTX equivalents/g as the limit value in Japan (reviewed by [2]). This toxin is not included in regular monitoring programs, which could pose a concern for public health, especially when there is a lack of occurrence and consumption data for marine gastropods, inhibiting the performance of a complete risk characterization. Thus, more data on TTX occurrence in bivalves and gastropods are required in order to establish meaningful regulations. 

The present study aims to evaluate the occurrence of TTX-contaminated bivalves and gastropods in marine environments and areas near the mouths of rivers in different points along the Spanish coast: Galicia, in the northwest (NW); Catalonia, in the northeast (NE); and Valencia, in the east (E), along different seasons of the year. This research was developed in the frame of a study focused on the possible effects of climate change on emerging risks affecting food safety by the presence of toxins not regulated at present in Europe. Spain is a referent region in Europe where bivalves are produced for human consumption, so the development of an LC-MS/MS method to detect and quantify TTX and its analogues will contribute to the assessment of the possible occurrence of TTX toxin in the estuaries close to sea harvesting areas. Taking into account that in UK and in the Netherlands contaminated samples were observed in the summer season, whereas in Greece positive samples were also detected at the end of the year [9], we focused the sampling in the NW of Spain (Atlantic) in June and September and in the E and NE (Mediterranean) in November–February.

## 2. Results

The samples analyzed include cultivated and uncultivated (referred as “wild”) molluscs. Species, origin and sampling place, date of harvesting and analytical results obtained are summarized in Table 1. None of the analyzed samples contained quantizable amounts of TTX. Indeed, no TTX peak under the LOQ (10 µg/kg TTX) was detected in any of the analyzed samples, although the Limit of Detection (LOD) was not determined.

## 3. Discussion

Few studies have been conducted on the presence of TTX in marine species other than fish from the Tetraodontidae family. Although TTX intoxication was traditionally considered to be confined to the warm waters of Asian countries, recent studies have reported the possible migration of different toxic fish species from the Red Sea to the Mediterranean Sea through the Suez Canal (reviewed in [1,12]). Regarding bivalves, this toxin, typically found in tropical latitudes, has been detected in the last years in a number of species from more temperate regions, such as the Mediterranean Sea [5], in bivalves from the United Kingdom [6,13] and in marine gastropods, among others, in New Zealand [14]. Concern about the occurrence of TTX in bivalves from temperate regions has triggered a number of studies globally, and high concentrations were detected in clams from New Zealand [15] as well as in mussels, oysters and hard clams from the UK [6,13,16]. TTX was also detected in mussels from Greece [5], in oysters and clams from the Netherlands [9], in clams from markets in China [17], as well as more recently in clams from New Zealand [18] and in very low concentrations in a single sample of cockle and oyster from Spain [7] and mussels and clams from Italy [8]. Regarding gastropods, in 2007 a first case of human intoxication with TTX with origin in European waters was reported. The intoxication was due to consumption of a trumpet shell (*Charonia lampas lampas*) caught in southern Portuguese waters [19].

As a consequence, attention has been paid to regulatory issues in the EU; the EFSA has recommended the evaluation of the presence of TTX in bivalves and gastropods from EU waters in order to perform an appropriate risk evaluation [9]. There are few publications on TTX occurrence in molluscs from the EU to date; therefore, we decided to perform a survey among different species of marine molluscs to provide data on this subject. This search included non-cultivated species like limpet, since they are traditionally consumed after recreational harvesting, or even some sea snails without commercial value, but which could transport the toxin indirectly to human consumers through the food web after being consumed by edible predators like crabs [20,21].

The results obtained in previous research on different bivalve molluscs from the Galician Rias on the west coast of Spain, where mussels in particular represent a very important resource, revealed low levels of TTX in only two samples out of 286 samples from infaunal areas, and at very low concentrations—2.3 μg/kg in cockles and below 0.9 μg/kg in oysters [7]. Further work developed in the same project did not find positive samples in 72 analyzed samples (cited in [22]). This study, together with a study on different intertidal molluscs and echinoderms from Portugal in which no bivalves positive for TTX were found (seven analyzed samples) [23], are the only studies regarding TTX in molluscs along the Iberian Peninsula. However, there are references to TTX in puffer fish (*Lagocephalus sceleratus*) caught along the Mediterranean coast of Spain [12].

The data on TTX occurrence in our study confirm these previous findings on bivalve molluscs, since our results are below the limit of quantitation (10 µg/kg) in all the samples, and thus lower than those recommended by the EFSA—44 μg TTX equivalent/kg of shellfish meat [9]. This reference value represents a standard currently applied by the Netherlands, the only EU country following the presence of TTX in shellfish, for measures to close production areas [24]. It has been reported that mainly the parent toxin is present in the bivalve toxin profiles [6,13], and this fact was confirmed by the results of Leão et al. (2018), who did not find TTX analogues in their samples [7]. Our results only refer to the presence of TTX; however, Turner et al. (2015) suggested that different TTX analogues could result from metabolism by shellfish, since only TTX and no analogues were found in *Vibrio* cultures isolated from molluscs which contained TTX and other analogues [6]. 

The samples in which Leao et al. (2018) found TTX were collected in February and June 2017 from intertidal areas, with water temperatures of 13.5 °C and 17 °C, respectively, and salinity levels around 35‰ [7]. Similarly, a study on the incidence of TTX in UK found positive samples throughout the year, although higher TTX concentrations were achieved in samples harvested in the warmer months of summer [13]. Shallow water, lower salinity, estuarine environments and water temperature over 15 °C were proposed as potential increasing risk factors [13]. Water temperatures and salinity were not recorded in this work, although samples were collected throughout the year. 

Concerning gastropods, some studies have suggested the bioaccumulation of TTX along the food-chain from gastropods (*Monodonta lineata, Gibbula umbilicalis, Nucella lapillus, Aplysia depilans, Pattela intermedia*) to echinoderms (*Marthasterias glacialis* and *Paracentrotus lividus*) (cited by [23]). Previous research on gastropods from the Portuguese coast reported some positive samples, collected in 2009 and 2010, containing amounts of TTX as well as other analogues such as 4-*epi*TTX, 5,6,11-trideoxyTTX and monodeoxyTTX (at low to moderate concentrations, in the range of 6–90 μg/kg) [23]. The authors reported that some of these gastropod species are consumed locally, directly harvested by people, so exposure to TTX and its analogues from gastropods is unknown and uncontrolled. The levels found by Silva et al. (2012) [23] were several orders of magnitude lower than those found during an intoxication incident in Malaga, Spain, in 2007, after consumption of a trumpet shell (*Charonia lampas*) collected in the south of Portugal, with a TTX concentration in the digestive gland of 315 μg/g and the variant 5,6,11-trideoxyTTX at a level of 1004 μg/g [19]. Our results correspond to gastropods collected in Galicia (*n* = 14), plus one sample of Purple dye murex from Catalonia. The TTX concentration was below 10 μg/kg; however, we should be cautious with these results, since other possible analogues were not studied and 5,6,11-trideoxyTTX was a major component in the 2007 episode in Málaga [19].

As in other marine biotoxins, it should be taken into account that TTX and its associated analogues are water-soluble and heat-stable, so they cannot be destroyed by cooking. So, they can cause serious poisoning and even death after ingestion. In addition, this problem has been increasing since the typical distribution of TTX in warm tropical waters seems to be changing with increasing sea water temperatures. As such, data on its occurrence in edible species is needed, as stated by the EFSA Contam Panel [9]. 

## 4. Conclusions

We analyzed different marine bivalves and gastropods collected from different places along the Spanish coast. TTX was not detected in any of the analyzed samples. Our results on TTX occurrence obtained in bivalve molluscs and gastropods indicate that TTX does not represent a risk for public health in Spain, at least with the data we have obtained so far. These results are in good accordance with results obtained previously in bivalves by other authors [7]. However, additional data on TTX occurrence should be obtained in specific areas and throughout the year in order to improve the risk assessment of TTX in marine bivalves and gastropods, following the EFSA’s recommendation.

## 5. Materials and Methods 

### 5.1. Reagents and Chemicals

All the reagents used for sample preparation, SPE (Solid Phase Extraction) and solvents and additives for HILIC-MS/MS were HPLC or LC-MS grade. Water HPLC-MS grade from VWR (Llinars del Vallés, Spain), acetonitrile LC-MS grade, glacial acetic acid (laboratory grade), formic acid eluent additive for LC-MS and ammonium hydroxide (solution 25% w/w, reagent grade) from Scharlab. (Sentmenat, Barcelona, Spain) Certified reference material (CRM) of tetrodotoxin (TTX) and 4,9-anhydro tetrodotoxin (4,9-anhTTX) was purchased from Cifga (Lugo, Spain).

### 5.2. Sampling

**Figure 1:**
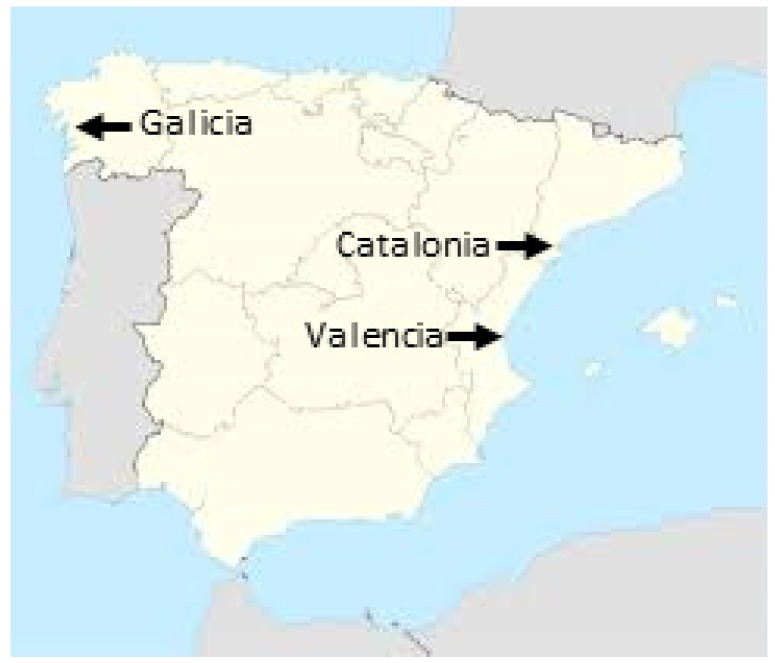
Location of sampling zones.

Samples were obtained from different sampling points along the Spanish coasts: mussels (*Mytilus galloprovincialis*) were obtained from several mussel raft cultures in Galicia (samples coming from eight different floating rafts in the Galician Rías), Catalonia (seven mussel samples) and Valencia (two mussel samples). Other sampled species were oysters (*Crassostrea gigas*) obtained from Catalonia (four samples) and Valencia (three samples); clams—*Donax* spp. obtained from Catalonia (one sample) and *Ruditapes* spp. from Galicia (one sample); and edible gastropods—*Bolinus* spp. from Catalonia (one sample). In addition, mussels and gastropods (six mussel samples attached to rocks; six samples of limpet, *Patella* spp.; eight samples of other gastropods) were sampled in several locations in Umia-O Grove, an intertidal area in the Ria of Arosa included in the Ramsar Convention for the conservation of wetlands. Samples were frozen at −20 °C until analysis. Sampling zones are depicted in Figure 1.

### 5.3. Extraction and Clean-Up

The extraction method described by the European Union Reference Laboratory for Marine Biotoxins (EU-RL-MB) [25] applied for the interlaboratory validation of HILIC-MS/MS for TTX in mussels was used in this study. This method proposed by Turner [16] for the validation of Paralytic Shellfish Poisoning (PSP) and TTX by HILIC-MS/MS was applied in the aforementioned reference, with slight modifications. Briefly, 5 mL 1% *v*/*v* acetic acid was added to 5.0 g of homogenized sample and thoroughly mixed on a vortex for 90 s, introduced in a boiling water bath for 5 min. After achieving room temperature and it was remixed on a vortex for 90 s and centrifuged (2500 rpm for 10 min). Then, 1 mL of the supernatant was transferred to a centrifuge tube and mixed on a vortex with 5 µL of 25% NH_3_, centrifuged at 10,000 rpm for 1 min and cleaned with Graphitized Carbon SPE (SPE-ENVI-Carb 250 mg/3 mL, Supelco, Sigma-Aldrich).

Clean-up was performed as follows: SPE cartridge was conditioned with 3 mL of a mixture of 20% acetonitrile and 1% acetic acid, followed by 3 mL of 0.025% *v*/*v* NH_3_ at 6 mL/min. Then, 400 µL of the sample extract were loaded at a flow of 6 mL/min, a washing step was performed with 700 µL of Mili-Q water at a flow of 6 mL/min and elution was carried out with 2 mL of a mixture of 20% acetonitrile and 1% acetic acid at a flow of 3 mL/min. Eluted extract was collected and mixed on a vortex. 100 µL of eluted extract were diluted with 300 µL of acetonitrile, mixed on a vortex and filtered through 0.22 µm filter to a polipropylene vial.

### 5.4. Standard Solutions

The commercial standard of TTX and 4,9-anhTTX (CRM-03-TTXs from Cifga, Lugo, Spain)—25.9 µg/mL TTX and 2.99 µg/mL 4,9-anhTTX in 1 mM acetic acid—was used to prepare a matrix matched 5 level calibration curve in the range 10–150 µg/kg TTX. These TTX standard solution extracts are stable for one week when preserved at 4 °C or less in polypropylene vials.

### 5.5. High-Performance Hydrophilic Interaction Liquid Chromatography-Tandem Mass Spectrometry (HILIC-MS/MS)

HILIC-MS/MS analyses of TTXs were carried out using a Waters (Milford, MA, USA) Xevo TQ-S tandem quadrupole mass spectrometer coupled to a Waters Acquity UPLC H-Class. 

A Waters ACQUITY UPLC Glycan BEH Amide Column, 130Å, 1.7 µm, 2.1 × 150 mm column was used. The analytical method was based on a method described by the EU-RL-MB and applied for the interlaboratory validation of HILIC-MS/MS for TTX in mussels, based on the method proposed by Turner [16] for the validation of PSP and TTX by HILIC-MS/MS. Chromatography conditions are described in Table 2. 

**Figure 2:**
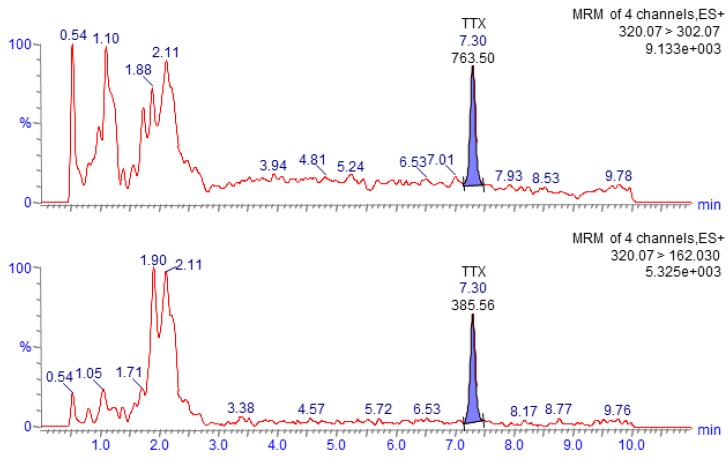
Chromatogram obtained after HILIC-MS/MS analysis of a matrix-matched standard tetrodotoxin (TTX) (10 µg/kg) representing the quantitation (*m*/*z* 320.07 > 302.07, upper) and confirmation (*m/z* 320.07 > 162.03, lower) MRM transitions.

The Waters Xevo TQ-S parameters were as follows: the capillary voltage was held at 3 kV, the desolvation temperature was 350 °C, the desolvation gas flow was 650 L/h, the polarity was positive and the MRM transitions are summarized in Table 3. The quantitation and confirmation *m/z* transitions used were 320.07 > 302.07 and 320.07 > 162.03, respectively. Figure 2 shows a chromatogram obtained following the analysis of TTX in a high-level calibration standard.

## Figures and Tables

**Table 1:** Analyzed samples and obtained results. LOQ: limit of quantitation. TTX: tetrodotoxin. ND: not detected.

Species	Location	Harvesting Date	Result (LOQ: 10 µg/kg TTX)
Clams *(Ruditapes decussatus)*	Ría of Arosa (NW)	12 June 2018	ND
Mussel (*Mytilus galloprovincialis*)	Valencia (E)	12 June 2018	ND
Mussel (*M. galloprovincialis*)	Valencia (E)	12 June 2018	ND
Mussel (*M. galloprovincialis*)	Ría of Arosa (NW)	15 June 2018	ND
Mussel (*M. galloprovincialis*)	Ría of Arosa (NW)	15 June 2018	ND
Mussel (*M. galloprovincialis*)	Ría of Arosa (NW)	15 June 2018	ND
Mussel (*M. galloprovincialis*)	Ría of Arosa (NW)	19 June 2018	ND
Mussel (*M. galloprovincialis*)	Ría of Arosa (NW)	19 June 2018	ND
Mussel (*M. galloprovincialis*)	Ría of Arosa (NW)	19 June 2018	ND
Mussel (*M. galloprovincialis*)	Ría of Arosa (NW)	20 June 2018	ND
Giant oyster (*Crassostrea gigas*)	Ebro Delta (NE)	24 December 2018	ND
Giant oyster (*C. gigas*)	Ebro Delta (NE)	24 December 2018	ND
Giant oyster (*C. gigas*)	Ebro Delta (NE)	31 December 2018	ND
Mussel (*M. galloprovincialis*)	Ebro Delta (NE)	15 January 2019	ND
Giant oyster (*C. gigas*)	Ebro Delta (NE)	15 January 2019	ND
Wild sea gastropod (*Calliostoma* spp.)	Umia-O Grove (NW)	28 September 2018	ND
Wild Mussel (*M. galloprovincialis*)	Umia-O Grove (NW)	28 September 2018	ND
Wild sea gastropod (*Calliostoma* spp.)	Umia-O Grove (NW)	28 September 2018	ND
Wild Mussel (*M. galloprovincialis*)	Umia-O Grove (NW)	28 September 2018	ND
Wild sea gastropod (*Calliostoma* spp.)	Umia-O Grove (NW)	28 September 2018	ND
Wild Mussel (*M. galloprovincialis*)	Umia-O Grove (NW)	28 September 2018	ND
Wild limpet (*Patella* spp.)	Umia-O Grove (NW)	28 September 2018	ND
Wild sea gastropod (*Calliostoma* spp.)	Umia-O Grove (NW)	7 September 2018	ND
Wild Mussel (*M. galloprovincialis*)	Umia-O Grove (NW)	7 September 2018	ND
Wild limpet (*Patella* spp.)	Umia-O Grove (NW)	7 September 2018	ND
Wild limpet (*Patella* spp.)	Umia-O Grove (NW)	7 September 2018	ND
Wild limpet (*Patella* spp.)	Umia-O Grove (NW)	7 September 2018	ND
Wild sea gastropod (*Calliostoma* spp.)	Umia-O Grove (NW)	7 September 2018	ND
Wild sea gastropod (*Calliostoma* spp.)	Umia-O Grove (NW)	7 September 2018	ND
Mussel (*M. galloprovincialis*)	Ebro Delta (NE)	18 February 2019	ND
Mussel (*M. galloprovincialis*)	Ebro Delta (NE)	18 February 2019	ND
Mussel (*M. galloprovincialis*)	Ebro Delta (NE)	18 February 2019	ND
Mussel (*M. galloprovincialis*)	Ebro Delta (NE)	18 February 2019	ND
Mussel (*M. galloprovincialis*)	Ebro Delta (NE)	25 February 2019	ND
Mussel (*M. galloprovincialis*)	Ría of Ares-Betanzos (NW)	27 February 2019	ND
Wedge clam (*Donax trunculus*)	Ebro Delta (NE)	26 February 2019	ND
Mussel (*Mytilus galloprovincialis*)	Ebro Delta (NE)	25 February 2019	ND
Giant oyster (*C. gigas*)	Valencia (E)	25 February 2019	ND
Giant oyster (*C. gigas*)	Valencia (E)	12 February 2019	ND
Giant oyster (*C. gigas*)	Valencia (E)	5 February 2019	ND
Wild limpet (*Patella* spp.)	Umia-O Grove (NW)	21 February 2019	ND
Wild mussel (*M. galloprovincialis*)	Umia-O Grove (NW)	21 February 2019	ND
Wild mussel (*M. galloprovincialis*)	Umia-O Grove (NW)	21 February 2019	ND
Wild limpet (*Patella* spp.)	Umia-O Grove (NW)	21 February 2019	ND
Wild sea gastropod (*Calliostoma* spp.)	Umia-O Grove (NW)	21 February 2019	ND
Wild sea gastropod (*Calliostoma* spp.)	Umia-O Grove (NW)	21 February 2019	ND
Purple dye murex (*Bolinus brandaris*)	Ebro Delta (NE)	5 March 2019	ND

**Table 2:** LC instrument conditions and high-performance hydrophilic interaction liquid chromatography-tandem mass spectrometry (HILIC-MS/MS) method.

Chromatographic Parameters			
Column	Waters ACQUITY UPLC Glycan BEH Amide Column, 130Å, 1.7 µm, 2.1 × 150 mm		
Injection volume	2 µL		
Column temperature	60 °C		
Sample temperature	4 °C		
Run time	11 min		
**Time (min)**	**Flow rate (mL/min)**	**% Mobile phase A1** Water–0.015% formic acid–0.06% ammonium hydroxide	**% Mobile phase B1** 70% Acetonitrile–0.01% formic acid
0	0.4	2	98
5	0.4	2	98
7.5	0.4	50	50
9	0.5	50	50
9.5	0.5	5	95
9.8	0.8	2	98
10.6	0.8	2	98
11	0.4	2	98

**Table 3:** MRM transitions used for the detection and quantitation of TTX.

Analyte	MRM Transitions	Cone, eV	CE, eV ^a^
TTX	320.07 > 302.07 ^b^; 320.07 > 162.03 ^c^	70 70	24 40

^a^ CE: collision energy; ^b^ quantitation MRM, ^c^ confirmation MRM.
